# The Relation Among Reactive Stepping and Fall-Related Psychological Factors in Multiple Sclerosis

**DOI:** 10.3390/brainsci14121197

**Published:** 2024-11-28

**Authors:** Taylor N. Takla, Patrick G. Monaghan, Daniel S. Peterson, Nora E. Fritz

**Affiliations:** 1 Neuroimaging and Neurorehabilitation Laboratory, Wayne State University, Detroit, MI 48201, USA; taylortakla@wayne.edu (T.N.T.); hr8036@wayne.edu (P.G.M.); 2 Translational Neuroscience Program, Wayne State University, Detroit, MI 48201, USA; 3 Department of Health Care Sciences, Wayne State University, Detroit, MI 48201, USA; 4 College of Health Solutions, Arizona State University, Phoenix, AZ 85004, USA; daniel.peterson1@asu.edu; 5 Department of Neurology, Wayne State University, Detroit, MI 48201, USA

**Keywords:** reactive stepping, falls, multiple sclerosis, concern about falling, balance confidence

## Abstract

**Purpose**: Persons with multiple sclerosis (MS) experience changes in balance, such as poor and reactive stepping, as well as altered fall-related psychological factors, such as increased concern about falling and feared consequences about falling. Such concerns and fear may relate to and influence mobility. However, these relations are poorly understood in people with MS. This study aimed to investigate the relation between reactive stepping performance and fall-related psychological factors, as well as to assess how these relations differ between individuals who have and have not fallen. **Methods**: In a single session, participants completed measures regarding fall-related psychological factors (balance confidence, concern about falling, and feared consequences of falling) and completed reactive stepping tasks. Following the visit, participants completed weekly surveys for 3 months to report their falls. Spearman rho correlations were computed to examine associations between participants’ reactive stepping performance and their fall-related concerns, confidence, and consequences, with a stratified analysis to compare these associations between fallers and non-fallers. **Results**: 44 individuals with MS participated in our study, with 27 individuals experiencing 0 falls (non-fallers) and 17 individuals experiencing at least 1 fall (fallers) in the 3-month follow-up period. Better reactive stepping performance was correlated with fewer concerns, greater confidence, and lower feared consequences related to falling. The stratified analysis revealed a greater number of significant associations for non-fallers than fallers, although the small sample of fallers reduced our ability to detect a relationship in this group. **Conclusions**: Reactive stepping was associated with fall-related psychological factors. Interventions targeting reactive stepping may be beneficial in enhancing fall-related psychological well-being in the MS community.

## 1. Introduction

Multiple Sclerosis (MS) is a chronic neurological disorder characterized by the demyelination of axons in the central nervous system, affecting approximately 2.5 million people worldwide [1,2]. One of the most prevalent and burdensome symptoms of MS is motor impairment, with over 85% of individuals with MS reporting some degree of motor dysfunction [3]. These motor impairments are a significant contributor to increased risk of falls in this population [4,5]. Falls are a common and serious concern for people with MS, with up to 60% experiencing at least one fall within a six-month period [6]. Beyond the immediate risk of physical injury, falls can lead to a cascade of adverse outcomes, including reduced physical activity, social isolation, diminished balance confidence, and decreased participation in daily activities, all of which negatively affect quality of life [7,8,9,10]. Given these consequences, addressing fall risk in individuals with MS is essential for developing effective interventions and improving long-term outcomes.

Reactive stepping, a crucial motor response for regaining stability after balance is lost, plays a vital role in fall prevention [11]. Reactive stepping involves recovering stability after unexpected perturbations and is distinct from standing balance, which focuses on maintaining stability during quiet stance or controlled movements. This distinction is crucial because most falls occur when individuals fail to recover from a perturbation, such as slips or trips. While traditional fall-prevention programs often target standing balance, reactive stepping deficits—strongly linked to fall risk in neurological populations—may require more tailored interventions in MS. Deficits in reactive stepping, namely delayed step initiation [12,13,14] and shorter step lengths [15], have been associated with increased fall risk in people with MS [16,17,18]. In fact, for every 10-millisecond delay in backward step initiation, individuals with MS are 2.5 times more likely to have a history of falls [18]. Reactive stepping deficits are not exclusive to individuals with MS and have been documented in other populations, such as older adults and individuals with Parkinson’s disease [19,20]. However, the underlying mechanisms contributing to these deficits may differ across populations. In MS, factors such as slowed neural conduction, proprioceptive impairments, and muscle weakness create unique challenges in reactive balance. Given the strong association between reactive stepping impairments and fall risk in MS, examining the specific balance recovery mechanism is critical to improving fall prevention strategies that may generalize across other clinical populations.

In addition to motor impairments, psychological factors related to falls, such as concern about falling (CAF), reduced balance confidence, and feared consequences of falling, are prevalent in MS [6,21]. Over 60% of individuals with MS report experiencing CAF [6,22], which has been linked to mobility limitations, as well as depression, anxiety, and avoidance behaviors [23,24,25]. Moreover, feared consequences of falling, which include worries about potential outcomes such as physical injuries, psychological distress, loss of independence, or social embarrassment, can lead to activity avoidance and diminished confidence in mobility. These factors are not merely consequences of falls but can also predict future fall risk [26,27]. For example, lower balance confidence has been associated with a higher likelihood of falls in people with MS [28], and when coupled with CAF, this can lead to activity avoidance or excessive caution. Paradoxically, these avoidance behaviors increase fall risk by promoting physical deconditioning, which further impairs balance and strength [21,23,29]. This intricate relation between fall-related psychological factors and motor impairments highlights the need for a comprehensive approach to improve both physical and mental well-being in MS, addressing both the motor and psychological components of balance and mobility.

While the association between fall-related psychological factors and overall fall risk in MS is well-established [6,21,23,27], there is limited understanding of whether these factors relate to reactive stepping performance [30,31]. Specifically, previous work indicates that increased threat (i.e., postural anxiety) could influence reactive balance [31], and CAF has been linked to poorer reactive balance in older women [30]. However, whether this relation extends to people with MS is unknown. Moreover, it remains unclear whether the relation between reactive balance and fall-related physiological factors differs between individuals with MS who have a history of falls and those who do not. This gap is critical, as reactive stepping is a crucial element of fall prevention, and understanding its relation with psychological factors could inform more targeted interventions.

Therefore, the purpose of this study was to investigate the relation between reactive stepping performance and fall-related psychological factors (balance confidence, CAF, and feared consequences of falling) in individuals with MS. A secondary aim was to determine whether this relation differs between those with and without a history of falls. Based on previous research associating greater fall risk with impaired reactive stepping [16,17,18] and elevated CAF [26,27], we hypothesized that greater CAF, lower balance confidence, and greater feared consequences of falling would be associated with poorer reactive stepping performance. Understanding these relations is crucial for informing more effective fall prevention strategies that address both motor and psychological factors, ultimately enhancing safety and quality of life for individuals with MS.

## 2. Materials and Methods

### 2.1. Participants

Participants were included if they met the following criteria: (a) had a diagnosis of relapsing-remitting MS using the MacDonald criteria [32]; (b) were between the ages of 18 and 65; (c) reported a Patient Determined Disease Steps (PDDS) ≤ 6, indicating ability to ambulate with or without an assistive device ≥ 50% of the time [33]; (d) have normal or corrected normal vision; and (e) were able to follow study-related commands. Participants were excluded if they (a) had an MS relapse in the past 8 weeks; (b) had an acute orthopedic condition that impacted walking (recent or sudden injuries or conditions that significantly impair mobility or balance, such as fractures, sprains); (c) had a comorbid neurological disorder; (d) used corticosteroids in the past 30 days; and (e) were currently taking dalfampridine or 4-Aminopyridine, a medication that may improve walking. Participants were recruited from the Wayne State University MS Center, the local chapter of the National Multiple Sclerosis Society, and a registry of individuals with MS who have expressed interest in research studies. If eligible, participants provided written informed consent before participating in the study, and the Wayne State University Institutional Review Board approved all study procedures.

### 2.2. Measures

During a single session, participants self-reported their demographic information (age, sex, race, disease severity [PDDS], symptom duration), filled out questionnaires regarding their concerns, confidence, and feared consequences related to falling, and participated in a reactive stepping task. Following the visit, participants received weekly surveys for 3 months to report the number of falls experienced that week [34,35]. Fallers were defined as individuals who experienced at least 1 fall in the 3-month period, and non-fallers were defined as individuals who experienced no falls during this prospective period.

### 2.3. Questionnaires

The Falls Efficacy Scale—International (FES-I) was used to measure CAF [36]. The FES-I is a 16-item questionnaire in which respondents rank their CAF through daily activities. A higher score represents greater CAF. The FES-I is a valid and commonly used questionnaire to assess CAF in persons with MS [37].

The Activities-specific Balance Confidence (ABC) Scale was used to measure participants’ confidence in performing various activities without losing their balance [38]. Respondents rate their confidence in performing specific activities on a scale from 0% (no confidence) to 100% (complete confidence). A higher score represents greater confidence in performing the activity without losing balance.

The Consequences of Falling Questionnaire (CoFQ) was used to assess participants’ feared consequences if they fell [39]. The CoFQ consists of items that fall into two sub-scales: items regarding the impact of falls on damage to one’s identity (e.g., feeling foolish, embarrassed) and items regarding the impact of falls on damage to one’s independence (e.g., becoming disabled, severely injured). Higher sub-scores represent greater feared consequences.

### 2.4. Instrumented Push and Release Test

The push and release test [40] was used to evaluate reactive balance performance. Prior to performing this test, participants were fitted with six APDM Opal wearable sensors (Opal, Generation 2, APDM, Inc., Portland, OR, USA), which allow for continuous recording and assessment of reactive balance performance. The sensors were placed on the top of the right and left feet, on the right and left wrists, at the lower lumbar vertebral level, and at the sternum. During the push and release test, participants began by standing comfortably with their feet shoulder-width apart. Research staff stood behind the participants and placed their hands on the participants’ scapulae. Participants were then instructed to lean back while keeping their feet flat on the ground, shifting their weight into the examiner’s palms. To deliver a consistent lean across all participants, administrators visually assessed the lean angle to ensure the midline of the participant’s body extended just beyond the participants’ heels in the sagittal plane. Once a stable leaned position was reached, the research staff abruptly removed their hands after a random interval between 2 and 5 s, necessitating the participants to take steps to restore their balance, and then freezing in place once balance was restored. If participants’ reactive stepping was insufficient to restore balance, the research team served as safety attendants to prevent any falls from occurring. Examiners collected clinical-grade measures of reactive stepping performance, which included counting the number of reactive steps participants took to regain their balance and rating their performance using a 5-point reactive step scale [41]. Only backward steps involved in regaining balance were included in the total step count; other steps that participants may have taken, such as realignment steps, were not included. Participants who took 0 steps were excluded from all analyses involving the number of steps, as 0 steps indicates an inability to initiate a reactive step, rather than a reflection of an attempt at balance recovery. For the clinical rating scale, lower scores indicate better reactive balance, reflecting the need for fewer steps to restore balance and the ability to recover independently (see Table 1) [41]. APDM sensors provided sensor-grade measures of reactive stepping performance [42], including postural latency, step latency, time to stabilize, and step length (Table 1). A total of five push and release trials were performed. The first two trials were used as familiarization, while the final three trails constituted the actual assessments. Therefore, reactive stepping outcome measures were averaged across the final three trials. Custom MATLAB algorithms were adapted from El-Gohary and colleagues [42] and were developed to analyze inertial sensor data and detect key events during the instrumented push and release assessment. Acceleration and rotational velocity data, sampled at 128 Hz, were processed to identify specific events throughout the task. These algorithms have previously been validated as reliable measures of postural responses in individuals with MS [42], and similar methods have been applied in studies on concussion and traumatic brain injury [43,44,45].

### 2.5. Mini Balance Evaluation Systems Test (Mini-BEST)

The mini-BEST reactive postural control sub-section was used as an additional clinical-based measure of reactive balance [40,46]. The mini-BEST is a clinical balance assessment used in persons with MS to evaluate anticipatory posture, reactive postural control, sensory orientation, and dynamic gait. Only performance on the reactive postural control section was used for analysis. In this section, participants perform the push and release test in the forward, backward, and sideways (left and right) directions. Each direction is scored on a scale from 0 to 2. Scores for the forward, backward, and sideways direction are summed to calculate the mini-BEST reactive postural control sub-score, using the sideways direction with the worst performance. Sub-scores range from 0 to 6, where higher scores indicate better reactive balance.

### 2.6. Statistical Analyses

Data analyses were conducted in SPSS V 29. Spearman rho correlations were computed to explore the association between participants’ reactive stepping performance and their concerns (FES-I), confidence (ABC Scale), and feared consequences (CoFQ) of falling. A stratified correlation analysis was performed to examine how the associations between reactive stepping outcomes and fall-related psychological factors varied between fallers and non-fallers. Correlation coefficients were interpreted as weak (0.1), moderate (0.3), or strong (0.5) [47]. Significance testing across all analyses was evaluated at *α* = 0.05.

## 3. Results

### 3.1. Sample Size and Demographics

A total of 44 participants (35F, 9M) with RRMS were recruited for participation. In total, 27 individuals experienced 0 falls in the three month monitoring period, whereas 17 individuals experienced at least 1 fall in the three months following the laboratory visit. Individuals who fell experienced a median of 2 falls in this three-month period, with the number of falls ranging from 1 to 24 (mean = 4.12, SD = 6.0). All participants were between the ages of 31–65. See Table 2 for a complete demographic profile of the sample.

### 3.2. Better Reactive Stepping Was Associated with Fewer Concerns, Greater Confidence, and Lower Feared Consequences Related to Falling

Reactive stepping performance was moderately to strongly correlated with CAF (Table 3). In particular, a faster time to stabilize (*r* = 0.499, *p* < 0.001), lower number of steps (*r* = 0.412, *p* = 0.006), better clinical rating scores (*r* = 0.499, *p* < 0.001), and better mini-BEST reactive postural control scores (*r* = −0.519, *p* < 0.001) were significantly correlated to lower CAF. Additionally, better clinical rating scores on the push and release (*r* = −0.363, *p* = 0.020) and mini-BEST reactive postural control scores (*r* = 0.324, *p* = 0.039) were moderately associated with greater balance confidence. Finally, time to stabilize (*r* = 0.322, *p* = 0.042) was moderately correlated with feared consequences of falling related to loss of independence. No other significant correlations were demonstrated among the measures.

**Figure 1:**
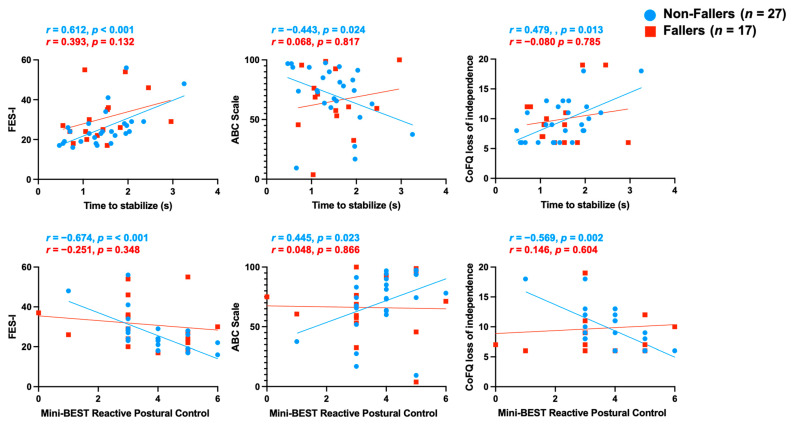
Scatterplots depicting bivariate relations between fall-related psychological parameters and reactive stepping outcome measures. *Note:* Spearman rho (*r*) correlation and corresponding *p* values for FES-I, balance confidence, and feared consequences of falling with time to stabilize and mini-BEST reactive postural control. Blue circles indicate non-fallers, and red squares indicate fallers.

In general, fallers reported greater CAF and less balance confidence (Table 2), but were not significantly different from non-fallers. The stratified correlation analysis based on fall status indicated strong to moderate correlations between reactive stepping and fall-related psychological parameters for non-fallers, while the faller group exhibited non-significant correlations (Figure 1). Specifically, time to stabilize, number of steps, clinical rating scores, and mini-BEST reactive postural control scores moderately to strongly correlated with concerns, confidence, and consequences relating to falling in the non-fallers, whereas no significant correlations were found in the fallers (Table 4). In the non-fallers, reactive stepping measures were most strongly correlated with CAF compared to confidence and feared consequences relating to falling. Specifically, lower CAF was strongly correlated with a faster time to stabilize (*r* = 0.612, *p* < 0.001), lower number of steps (*r* = 0.493, *p* = 0.009), better clinical rating scores (*r* = 0.578, *p* = 0.002), and better mini-BEST reactive postural control scores (*r* = −0.674, *p* < 0.001). All other reactive stepping measures exhibited no significant correlations.

## 4. Discussion

This study aimed to investigate the relation between reactive stepping performance and fall-related psychological factors in MS. While previous research has shown that fall-related psychological parameters negatively impact balance control in older women [30] and healthy young adults [31], our findings extend this work by establishing this relation in an MS population. We hypothesized that greater CAF, lower balance confidence, and greater feared consequences of falling would correlate with poorer reactive stepping performance. Our findings partially support this hypothesis, revealing significant associations between negative fall-related psychological factors and poorer reactive stepping performance. Specifically, we found significant correlations among greater CAF, lower balance confidence, and greater feared consequences of falling with a longer time to stabilize, greater number of steps, and worse clinical ratings during the push and release test, and poorer scores on the mini-BEST reactive postural control section. These correlations were more pronounced in non-fallers compared to fallers, however further investigation in larger samples is necessary to draw definitive conclusions about the strength of these relations. It is particularly important to acknowledge that the smaller sample size for the faller group may limit the ability to make robust conclusions regarding these correlations. Nevertheless, this underscores the importance of addressing both motor and psychological components in fall prevention strategies for individuals with MS who have and have not fallen yet.

Our findings revealed more significant associations between reactive stepping performance and fall-related psychological factors in MS non-fallers compared to fallers. One potential reason for this difference could be the wide variability in performance among the fallers in the reactive stepping assessments and self-report measures (Figure 1). Moreover, it is plausible that reactive stepping abilities in fallers may be more influenced by various other factors, including their motor impairments and proprioception [13,48]. For example, prior work from our lab has shown that proprioception is a greater predictor of backward walking compared to forward walking performance [49], highlighting the sensory demands of backward movements such as reactive backward stepping. Further, this prior work also highlighted that MS fallers had worse proprioception compared to MS non-fallers [49], highlighting the critical role of proprioception in balance maintenance [50]. Therefore, the findings suggest that the relation between reactive stepping and fall-related psychological factors may be more complex in MS fallers, potentially due to the influence of additional sensory and motor impairments. These differences highlight the need for a nuanced understanding of how psychological factors interact with physical capabilities in individuals with MS, particularly in developing interventions to address the unique needs of both fallers and non-fallers. However, these findings should be interpreted cautiously due to the limited sample size, overall mild disease severity, and short prospective fall monitoring period. Further, non-fallers were classified by not having fallen in a 3-month prospective period after testing, yet it is possible that these individuals have had prior falls that negatively impact their CAF or reactive postural control. Future larger-scale studies are needed to better understand the influence of fall status on the relation between reactive stepping and fall-related psychological factors across the full spectrum of MS-related disability. Exploring the specific contributions of sensory, motor, and psychological factors may help elucidate the underlying mechanisms driving reactive stepping performance and fall risk in MS.

An interesting observation from our findings was that we report significant correlations between both clinical-grade and sensor-based measures of reactive stepping with fall-related psychological factors. While sensor-based measures require specialized equipment and complex data analysis, clinical-grade measures can be easily collected in a standard clinical setting. This observation holds important implications for scalability to clinical practice, suggesting that clinical-grade measures may offer a practical and accessible approach for assessing reactive balance in MS, while also relating to patients’ feelings regarding fall experiences. Given their ease of collection, alongside their moderate to strong correlations with patients’ beliefs about CAF, balance confidence, and feared consequences of falling, clinical-grade measures are well-suited for developing treatment plans that address both the physical and psychological dimensions of fall risk. Therefore, clinical-grade measures of reactive balance should be considered alongside sensor-based assessments, as they provide feasible and effective means to capture valuable insights into fall risk and patients’ perceptions.

The use of clinical-grade measures does not diminish the potential utility of sensor-based measures. For instance, while most sensor-grade measures (step latency, postural latency, and step length) did not show significant correlations with fall-related psychological factors, time to stabilize demonstrated significant associations with CAF and feared consequences of falling across the sample (Table 3), along with balance confidence in the non-fallers (Table 4). Prior work has shown that with longer latency responses to perturbations, cortical involvement increases, whereas short latency responses are primarily mediated by the spinal cord and brainstem [51]. This suggests that time to stabilize, a longer latency response compared to the other sensor-grade measures, may demand greater cortical involvement than step and postural latency, reflecting its association to fall-related psychological parameters. Since time to stabilize measures the duration from the time of release until full recovery of balance, this extended period likely allows individuals more time to assess the situation and recognize the potential risk of falling, leading to heightened cognitive and emotional responses. In contrast, postural and step latencies only measure the interval from the time of release to the initiation of the first step or the first heel strike, respectively. These rapid responses provide less opportunity for individuals to acknowledge potential fall risks, making them less influenced by fall-related psychological beliefs, such as CAF. Consequently, while time to stabilize may be associated with a conscious awareness of risks, the shorter measures of postural and step latency may operate without this higher-level cognitive engagement, explaining their lack of significant correlations with psychological factors.

Overall, while clinical-grade measures provide practical and feasible insights into the link between physical performance and psychological factors, sensor-based measures may still hold value in capturing specific nuances of reactive balance control. We believe these measures can provide complementary insights, as sensor-grade outcomes offer more detailed, objective data on balance and stepping performance, potentially identifying fall risk earlier in individuals where clinical measures may fall short. This dual approach could lead to more personalized and effective fall prevention interventions. A combinatory approach using both clinical and sensor-based assessments may therefore be most effective, leveraging the complementary strengths of each method to gain a comprehensive assessment of reactive stepping. What remains unclear is the responsiveness to change of these measures to rehabilitation programs targeting reactive balance control. Future work should examine the responsiveness to change of both clinical-grade and sensor-based measures to better understand how these tools can effectively track progress and inform treatment strategies in MS.

### Limitations

This study has several limitations that should be acknowledged. First, our sample was restricted to individuals with relapsing-remitting MS, which limits the generalizability of the findings to those with progressive forms of MS. Additionally, the largely female low-disability sample (mean PDDS = 1.93) may limit the generalizability to males and those with greater disease severity. Moreover, while this study focused specifically on individuals with MS and excluded those with other neurological disorders or health conditions affecting gait, we did not examine the potential influence of additional factors such as comorbid health conditions, medication use, or other demographic variables (e.g., assistive device use, disease severity) on fall risk or reactive stepping performance. Additional motor and sensory functions were not examined within the scope of the current study; however, they may warrant further investigation to assess their potential impact on reactive stepping outcomes. These unexamined factors may have influenced the findings and should be considered when interpreting the findings. Another potential limitation of this study is the possibility that, despite instructions to freeze and remain stable after regaining balance, some participants may have taken an additional step at the end of the push and release task. However, we mitigated this by using the last three trials to ensure participants were fully familiarized with the task instructions, reducing the likelihood of extraneous steps affecting the reactive balance outcomes. Furthermore, the relatively small sample size of 44 participants, including only 17 fallers, may constrain the statistical power to detect significant associations. Finally, the 3-month monitoring period may not adequately capture the full spectrum of fall events, particularly in individuals with relapsing-remitting MS who experience fluctuations in their symptoms, although studies have shown that 54% of individuals with MS have fallen within a 3-month period [52]. This 3-month prospective period also does not capture persons with MS who may have experienced prior falls that impact their baseline performance. Future research should involve both a detailed fall history as well as a longer prospective fall monitoring period with a larger and more diverse sample size. Additionally, future studies should examine the impact of targeted interventions on the relation between reactive stepping and these fall-related psychological factors. Such studies may provide valuable insights into effective fall prevention strategies that also improve psychological well-being in the MS community.

## 5. Conclusions

This study identified a novel link between reactive stepping performance and fall-related psychological factors in individuals with MS. Our findings revealed that greater CAF, lower balance confidence, and greater feared consequences of falling were associated with poorer reactive stepping outcomes, particularly in non-fallers. Our results lay the foundation for future work exploring the need for a dual approach in fall prevention strategies that address both motor and psychological dimensions of balance recovery. Ultimately, further understanding these relations will inform targeted interventions that enhance both physical performance and psychological well-being, thereby improving the overall quality of life for the MS community.

## Figures and Tables

**Table 1:** Definitions of clinical and sensor-grade reactive balance outcomes.

Outcome Measure	Definition
**Clinical-Grade Measures of Reactive Balance**	
Clinical Reactive Step Rating	The number of reactive steps participants took to regain their balance and performance rating using a 5-point push and release scale [41]: (0) = recovers independently with 1 step, (1) = two or three small steps backward, but recovers independently, (2) = four or more steps backward but recovers independently, (3) = steps but needs assistance to prevent a fall, (4) = falls without attempting a step or unable to stand without assistance. Lower scores on this scale indicate better reactive balance performance.
Total Number of Reactive Steps	The number of backward reactive steps participants took to regain balance upon release of support. Non-reactive steps, such as realignment steps, were not included in the total step count.
Mini-BEST Reactive Postural Control	Participants perform a push and release task in the forward, backward, right, and left direction. Each task is rated on a 0–2 scale. (0) = Severe: no step, or would fall if not caught, or falls spontaneously. (1) = Moderate: more than one step used to recover equilibrium. (2) = Normal: recovers independently with a single, large step. Only the lowest score from the left or right is used in scoring. The maximum score is 6, with higher scores indicating better reactive balance performance.
**Sensor-Grade Measures of Reactive Balance**	
Postural Latency (s)	Time from release to the initiation of the first step, identified when foot acceleration exceeded 7% of gravity and the rotational rate surpassed 7°/s.
Step Latency (s)	Time from release to the first heel strike, determined when total foot acceleration exceeded 18 m/s^2^.
Time to Stabilize (s)	Time from release to when the trunk became stationary; was detected when lumbar sensor acceleration dropped below 7% of gravity and the rotational rate fell below 7°.
Step Length (m)	Length of the first step was calculated by taking the double integral of the foot’s acceleration in the anterior–posterior plane.

Note: Definitions of sensor-grade measures of reactive balance were adapted from El-Gohary 2017 [42]. Representative acceleration data from push and release trials can be found in Supplementary Figure S1, adapted from Morris 2020 [44].

**Table 2:** Description of sample demographics.

Variable	Entire Sample	Non-Fallers	Fallers	*p*-Value
Sample Size, *n*	44	27	17	
Female, *n* (%)	35 (79.5%)	22 (81.5%)	13 (76.5%)	
Age (years)	47.77 ± 9.86	47.07 ± 9.91	48.88 ± 9.98	0.560
Symptom Duration (years)	14.02 ± 9.90	13.37 ± 10.63	15.06 ± 8.82	0.588
Disease Severity (PDDS)	1.93 ± 2.02	1.37 ± 1.67	2.82 ± 2.24	**0.029**
Race, *n* (%)	Black or African American: 22 (50.0%) White: 19 (43.2%) Pacific Islander: 1 (2.3%) Biracial or Multiracial: 2 (4.5%)	Black or African American: 15 (55.6%) White: 9 (33.3%) Pacific Islander: 1 (3.7%) Biracial or Multiracial: 2 (7.4%)	Black or African American: 7 (41.2%) White: 10 (58.8%)	
Postural Latency (seconds)	0.21 ± 0.07	0.20 ± 0.06	0.24 ± 0.08	0.109
Step Latency (seconds)	0.53 ± 0.11	0.51 ± 0.10	0.57 ± 0.12	0.098
Time to Stabilize (seconds)	1.44 ± 0.63	1.45 ± 0.64	1.43 ± 0.64	0.926
Step Length (meters)	0.22 ± 0.14	0.19 ± 0.12	0.26 ± 0.15	0.087
Number of Steps	2.48 ± 1.39	2.53 ± 1.34	2.4 ± 1.39	0.780
Clinical Rating of Reactive Postural Control	1.23 ± 0.99	1.12 ± 0.80	1.39 ± 1.24	0.386
Mini-BEST Reactive Postural Control	3.81 ± 1.30	3.96 ± 1.09	3.56 ± 1.59	0.333
FES-I	27.75 ± 10.52	25.78 ± 9.48	30.88 ± 11.60	0.118
ABC Scale	69.05 ± 25.14	70.79 ± 24.75	66.04 ± 26.39	0.567
CoFQ Functional Independence	9.63 ± 3.78	9.58 ± 3.51	9.73 ± 4.33	0.900
CoFQ Damage to Identity	13.10 ± 4.31	13.19 ± 4.22	12.93 ± 4.61	0.856

Note: Demographic profile is reported for entire sample and was stratified by fall status. Sample means and standard deviations are reported (M ± SD) unless otherwise noted. Bolded *p*-values indicate significant group differences between fallers and non-fallers.

**Table 3:** Bivariate correlations among reactive stepping and fall-related psychological factors across the entire sample (*N* = 44).

	FES-I	ABC Scale	CoFQ Functional Independence	CoFQ Damage to Identity
Postural Latency	0.194	−0.148	−0.079	−0.243
Step Latency	0.144	−0.085	−0.242	−0.090
Time to Stabilize	0.499 **	−0.254	0.322 *	0.126
Step Length	0.121	−0.020	−0.009	0.168
Number of Steps	0.379 *	−0.197	0.333 *	0.207
Clinical Rating	0.499 **	−0.363 *	0.170	0.087
Mini-BEST Reactive Postural Control	−0.519 **	0.324 *	−0.286	−0.256

Note: Spearman rho correlations are reported for the bivariate relation between fall-related psychological parameters and reactive stepping performance indexed by various sensor-based and clinical-based measures. Significant values are indicated in bold. * *p* < 0.05, ** *p* < 0.01.

**Table 4:** Bivariate correlations among reactive stepping and fall-related psychological factors stratified by fall status.

	Non-Fallers (*n* = 27)				Fallers (*n* = 17)			
	FES-I	ABC Scale	CoFQ Loss of Independence	CoFQ Damage to Identity	FES-I	ABC Scale	CoFQ Loss of Independence	CoFQ Damage to Identity
Postural Latency	−0.032	−0.174	−0.033	−0.299	0.454	−0.128	−0.191	−0.203
Step Latency	0.014	−0.058	−0.203	0.024	0.343	−0.108	−0.326	−0.315
Time to Stabilize	0.612 **	−0.443 *	0.479 *	0.253	0.393	0.068	−0.080	−0.049
Step Length (x)	0.236	−0.042	−0.142	0.052	−0.047	−0.007	0.254	0.295
Number of Steps	0.442 *	−0.262	0.516 **	0.292	0.308	−0.089	−0.114	0.120
Clinical Rating	0.578 **	−0.515 **	0.448 *	0.246	0.373	−0.111	−0.265	−0.090
Mini-BEST Reactive Postural Control	−0.674 **	0.445 *	−0.569 **	−0.435 *	−0.251	0.048	0.146	−0.047

Note: Spearman rho correlations are reported for the bivariate relation between fall-related psychological parameters and reactive stepping performance indexed by various sensor-based and clinical-based measures for both non-fallers and fallers. Significant values are indicated in bold. * *p* < 0.05, ** *p* < 0.01.

## Data Availability

The data that support the findings of this study are not openly available due to reasons of sensitivity and are available from the corresponding author upon reasonable request.

## Supplementary Materials

The following supporting information can be downloaded at https://www.mdpi.com/article/10.3390/brainsci14121197/s1, Figure S1: Representative acceleration data from the push and release trial, adapted from Morris 2020 [44]. Support was released at time 0 (solid yellow line). Step latency (blue dashed line) and stabilization time (yellow dashed line) are indicated.
