# Supplementary material for: The Relation Among Reactive Stepping and Fall-Related Psychological Factors in Multiple Sclerosis
Source: Brain Sci. 2024 Nov 28;14(12):1197. doi: 10.3390/brainsci14121197 (PMC11674261; doi:10.3390/brainsci14121197)
Supplement: Supplementary file 1 [file brainsci-14-01197-s001.zip › brainsci-3320747-supplementary.pdf]

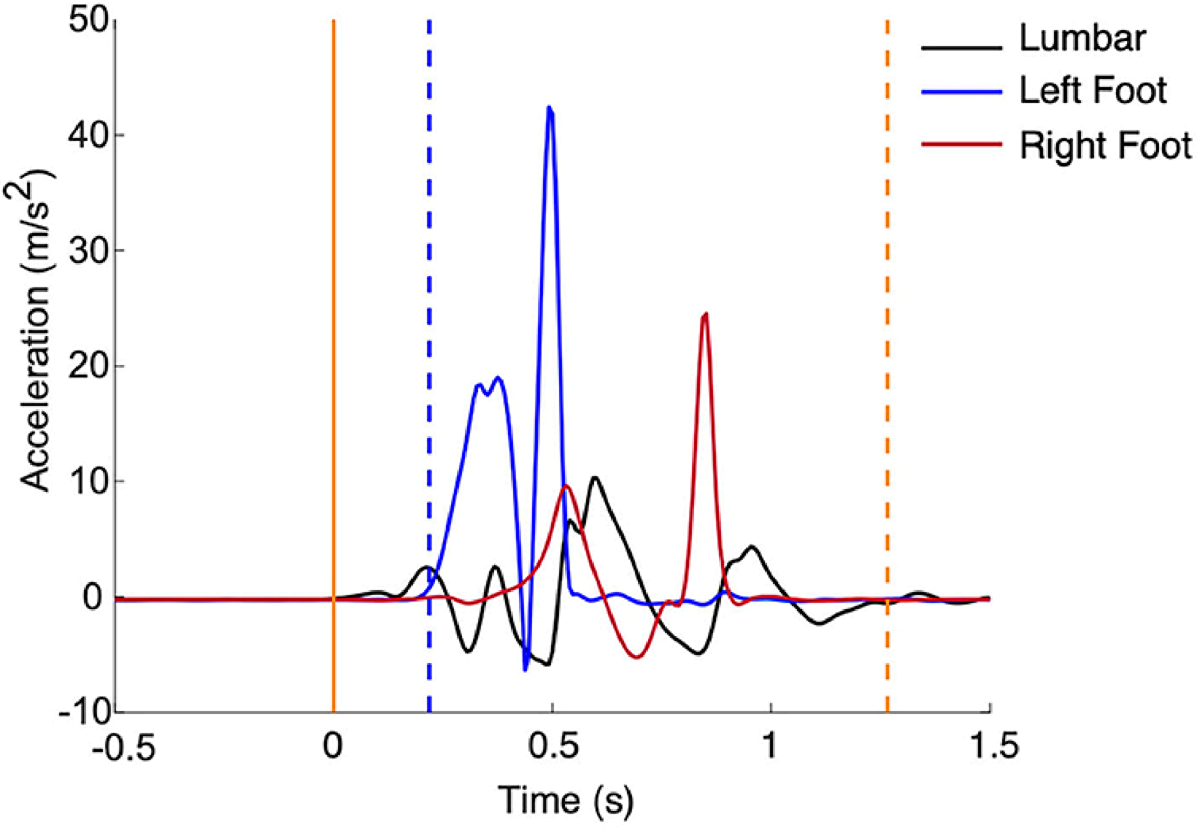

Supplementary Figure S1: Representative acceleration data from the push and release trail, adapted from Morris 2020 [44]. Support was released at time 0 (solid yellow line). Step latency (blue dashed line) and stabilization time (yellow dashed line) are indicated.
